# Research advances of the establishment and characterization of *Helicobacter pylori* infection animal models

**DOI:** 10.3389/fmicb.2025.1683366

**Published:** 2025-10-15

**Authors:** Kaifang Zhang, Man Cui, Yuefeng Zhu, Songping Li, Guimin Su, Lin Du

**Affiliations:** ^1^School of Life Science and Biopharmaceutics, Shenyang Pharmaceutical University, Shenyang, China; ^2^Beijing Zhifei lvzhu Biopharmaceutical Co., Ltd., Beijing, China; ^3^Beijing Bacterial Vaccine Engineering Research Centre, Beijing, China

**Keywords:** *Helicobacter pylori*, animal model, infection model, establishment methods, animal species selection, detection, quantification

## Abstract

*Helicobacter pylori* (Hp) is a major pathogenic bacterium responsible for gastritis, peptic ulcers, and gastric cancer. The prevention and control of Hp infection pose significant global health challenges, particularly due to the rising antibiotic resistance alongside the current absence of an effective vaccine. This review synthesizes the key elements governing successful model establishment, including strain characteristics, animal host species selection, pre-treatment methods, and infection protocols. It further elaborates on the methods for detecting and quantifying Hp in animal models, including invasive and non-invasive detection techniques for model validation, and explores the potential applications of spatial transcriptomics in this field. Furthermore, the review outlines current progress and limitations in Hp infection modeling. Aimed at supporting preclinical research, this review serves as a practical reference for establishing stable and reproducible animal models that mimic human infection and disease progression, thereby facilitating the evaluation of novel therapeutics and vaccine candidates.

## 1 Introduction

*Helicobacter pylori* (Hp), a microaerophilic gram-negative bacterium, persistently colonizes in the human gastric mucosa. Hp infection has been widely recognized as a primary causative factor of chronic gastritis and peptic ulcers, and is closely associated with the development of gastric cancer (Ansari and Yamaoka, 2022). According to the latest GLOBOCAN data, the incidence and mortality rates of gastric cancer rank fifth among all malignant neoplasms (GLOBOCAN, 2022). A study based on GLOBOCAN 2022 country-specific age-standardized incidence rates and United Nations population projections revealed that among birth cohorts from 2008 to 2017, an estimated 15.6 million new gastric cancer cases are projected to occur worldwide, with approximately 76% attributable to Hp infection (Park et al., 2025).

While conventional antibiotic therapy demonstrates effectiveness, it still faces several major limitations, including poor compliance due to side effects, rising failure from resistance, high costs, and no durable protection against reinfection (Mascellino et al., 2022; Medakina et al., 2023). In recent years, the continuous increase in antibiotic resistance has posed a persistent threat to human health. Given this severe situation, the development of vaccines has become a key approach to addressing this issue.

Hp has coevolved with its human host over thousands of years, enabling it to establish chronic infection through multifaceted immune evasion strategies. These include molecular mimicry of host antigens and antigenic variation to circumvent immune recognition (Moxon and Thaler, 1997; Maldonado et al., 2016; Dunne et al., 2014). The bacterium also secretes key virulence factors that suppress immune cell function and foster an immunosuppressive microenvironment (Viala et al., 2004; Lu et al., 2021; Hu et al., 2025). Due to the immune evasion mechanisms of Hp, interventions that prove effective in animal models often fail in human clinical trials (Sutton and Boag, 2019; Sutton and Doidge, 2003; Sutton and Chionh, 2013). This highlights fundamental limitations of existing animal models in recapitulating the complexity of human infection, such as inconsistent success rates and difficulties in reliably reproducing key features like colonization, chronic inflammation, and pathological progression. Therefore, there is an urgent need to develop stable, reproducible, and highly mimetic animal models that accurately reflect human infection.

In this context, this review focuses on the establishment of Hp infection models and the detection and quantification of the bacterium within these models. This review provides a comprehensive overview of the key elements involved in establishing Hp infection animal models and summarizes recent advances in model detection techniques. The aim is to provide a practical reference for establishing stable, reproducible, and highly human-relevant animal models that support the preclinical evaluation of novel therapeutic agents and candidate vaccines.

## 2 Key elements in animal model establishment

**Figure 1:**
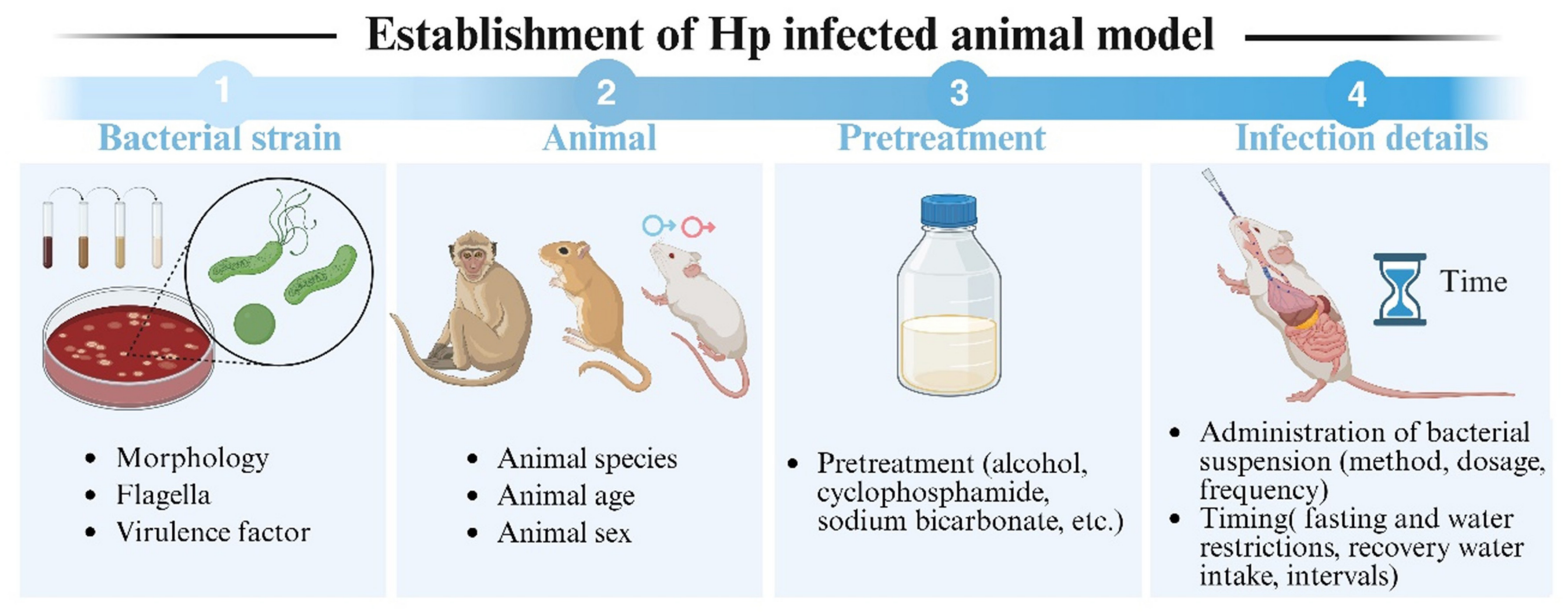
Schematic diagram of the establishment of an Hp infection animal model. This figure illustrates the factors to be involved in establishing an Hp infection model, including strain characteristics (such as morphology, species, flagella, and virulence factors), animal species, pre-treatment, and details of the infection.

Establishing reliable Hp animal models faces multidimensional challenges, necessitating comprehensive optimization of strain characterization (accounting for morphology, flagellar, and strain heterogeneity), host species, pre-treatment and infection procedure (Figure 1). These efforts aim to enhance both the success rate and reproducibility of model construction.

### 2.1 *Helicobacter pylori* strains characteristics

#### 2.1.1 Morphology

Morphological changes in Hp impair its ability to colonize, with its helical shape being critical for the bacterium's colonization in the stomach (Sycuro et al., 2010, 2012, 2013). Genes *csd1-6* and *ccmA* regulate peptidoglycan cross-linking and pruning to maintain the helical shape of Hp. Among these, knockout mutant strains of *csd1, csd3*, and *csd4* exhibited reductions in motility of 11%, 25%, and 17% in semi-solid agar, respectively. Further comparative studies indicated that the *csd3* mutant strain demonstrated a significantly reduced colonization efficiency in mice after gavage compared to the wild-type Hp (Sycuro et al., 2012; Krzyżek and Gościniak, 2018; An et al., 2015). Live cell tracking techniques were employed to investigate how helical shape affects Hp motility in mucus, demonstrating that the helical wild strain exhibited a 7–21% increase in swimming speed compared to the straight-rod mutant (Salama, 2020), along with a 15% enhancement in propulsive force (Constantino et al., 2016). Thus, the helical shape enables Hp to navigate efficiently in the gastric mucosa and to penetrate mucus for colonization.

Under environmental stresses such as antibiotic exposure or nutritional deprivation, Hp can transform into a spherical coccoid form. This morphological transition is mediated by the type I toxin-antitoxin system, which influences bacterial growth and division processes, inducing the shift from helical to coccoid morphology (Cheng and Boneca, 2024; Mao et al., 2021). Additionally, the deletion of genes encoding lipopolysaccharide glycosyltransferase has been shown to trigger a similar morphological shift from helical to coccoid forms (Tang et al., 2023). Although coccoid Hp exhibits reduced pathogenicity compared to its helical counterpart, it retains the ability to produce urease and adhere to epithelial cells, thus maintaining its capacity to induce gastritis (She et al., 2003). Chaput et al. (2006) further confirmed that the spherical variant can evade recognition by the human innate immune receptor Nod1, inducing neither NF-κB activation in HEK 293T cells nor IL-8 production in gastric epithelial cells. In the animal study, She et al. (2003) demonstrated that the positive rate of the gastric mucosa urease test was significantly lower in the coccoid Hp-infected group (50%) than in the spiral form-infected group (93.8%). Meanwhile, the culture positivity rate of coccoid Hp (68.8%) was also lower compared to the spiral form-infected group (87.5%).

#### 2.1.2 Flagella

Hp possesses 4 to 8 unipolar flagella that play critical roles in bacterial colonization and the induction of inflammatory responses. These flagella are enveloped by a specialized sheath that provides protection against gastric acidity, thereby ensuring their structural integrity in the harsh gastric environment (Geis et al., 1993). Flagella-mediated motility is essential for Hp to colonize the paramucosal mucus layer adjacent to the gastric epithelium, which constitutes the pathogen's ecological niche (Eaton and Krakowka, 1994; Eaton et al., 1992). The pivotal role of motility in colonization was first demonstrated by Eaton et al., whose study suggested that motile strains exhibited significantly higher infection rates and maintained sustained colonization advantages in germ-free piglet models (Eaton et al., 1992). This observation has been corroborated by multiple animal studies utilizing motility-deficient mutants. For instance, *FlgE*-deficient strains exhibit a complete loss of motility (O'Toole et al., 1994), while the dual deletion of flagella filament components *FlaA* and *FlaB* not only disrupts flagella ultrastructure but also renders the bacteria avirulent, keeping it from colonizing even after prolonged exposure in animal models (Eaton et al., 1996; Josenhans et al., 1995).

The most compelling evidence comes from the research by Osaki et al., which found that changes in flagellin glycosylation significantly affect the motility of the strains. Specifically, as the glycosylation level of the FlaA protein increases, both the motility and colonization ability of the strains are elevated (Osaki et al., 2006). Notably, although the *FliD* mutant strain can form short flagella, it completely loses the ability to penetrate the gastric mucosa, rendering it incapable of colonizing the mouse gastric mucosa (Kim et al., 1999). Furthermore, the *motB* deleted strain maintains an intact flagellar structure; however, its colonization is reduced by more than 80% compared to the wild type due to the loss of proton motive force (Ottemann and Lowenthal, 2002).

#### 2.1.3 Strain heterogeneity

A prominent issue in constructing animal models is the lack of heterogeneity in the selection of challenge strains. Clinical isolates often exhibit poor colonization capacity in standard animal models, due to their adaptation to the human-specific environment, leading to widespread reliance on the Hp SS1 strain (Santos et al., 2011; Yanaka et al., 2009; Santos et al., 2015). Liu et al. summarized the commonly used Hp strains and compared their genes, virulence factors, and animal models of contagious infection (Liu et al., 2024). Hp cagA-positive strains (corresponding to strains that contain the cag pathogenicity island) are associated with a higher risk of gastric cancer or premalignant lesions than cagA-negative strains (Figueiredo et al., 2002; Blaser et al., 1995; Plummer et al., 2007). Similarly, Hp strains containing specific OMP-encoding genes are associated with an increased risk of gastric cancer or premalignant changes compared to strains that lack these genes or that harbor out-of-frame genes. Therefore, virulence factors are one of the factors influencing strain heterogeneity (Gerhard et al., 1999; Prinz et al., 2001; Yu et al., 2002; Yamaoka et al., 2006; Talebi Bezmin Abadi et al., 2011).

To enhance the clinical translation value of Hp vaccine research, it is essential to overcome the challenges of insufficient strain heterogeneity and low colonization efficiency of clinical isolates. First, Hp type I strains are defined as strains carrying an intact and functional cag pathogenicity island (cag PAI), expressing a functional T4SS and producing an active VacA. Hp type I strains are key factors in causing severe gastric diseases in humans, but they cannot establish stable infections in mouse models. Research has shown that gerbils can achieve stable colonization of Hp type I strains and facilitate pathogenicity studies (Watanabe et al., 1998; Peek et al., 2000; Rieder et al., 2005; Hoffelner et al., 2008). For vaccination research, the Mongolian gerbil model may serve as a robust alternative to the Hp mouse model. Second, subjecting clinical isolates to animal passage adaptation can significantly enhance their colonization capacity in models (Bleich et al., 2005). Furthermore, challenge experiments should prioritize the use of clinical isolates or engineered strains expressing key virulence factors (such as cag-PAI) (Rieder et al., 2005; Gangwer et al., 2010) and well-characterized strains adapted to specific animal models (e.g., the J166 strain isolated from rhesus monkeys, Solnick et al., 2001; Dubois et al., 1996) may also be considered. Through the integrated application of these strategies, a more accurate simulation of the immunopathological features of human infection might be achieved, thereby improving the predictive power of preclinical experiments.

### 2.2 Host species selection

#### 2.2.1 Mouse

Hp is a significant pathogen associated with various gastric diseases, and mouse infection models are crucial for investigating its infection mechanisms and pathophysiology. Lee et al. (1997) were the first to screen and establish a mouse-adapted standard strain of Hp SS1 (Sydney Strain 1) from the human clinical isolate 10,700 (PMSS1), which demonstrated colonization in C57BL/6 mice for more than 8 months and induced chronic gastritis and atrophy. Although significant progress has been made in the study of Hp SS1-infected mouse models, their application still has certain limitations. Firstly, there is considerable uncertainty in translating vaccine research findings from this model to clinical applications, as multiple vaccines that demonstrate protective effects in mice have failed to show efficacy in human clinical trials (Sutton and Boag, 2019). Furthermore, due to the poor stability of the T4SS in the mouse gastric environment, infection typically results only in mild gastritis and seldom progresses to more severe pathologies (such as peptic ulcers or gastric cancer) (van Doorn et al., 1999).

Although the SS1 infection model has limitations in mimicking severe human disease, the mouse model remains widely used for evaluating vaccine immunogenicity. Among these, BALB/c and C57BL/6 mice are the most widely used models for Hp immunization studies. Both species can mount comprehensive immune responses resembling those in humans through Th1, Th2, and Th17 pathways, yet each is preferred for distinct applications. BALB/c mice are often preferentially selected for the preclinical evaluation of recombinant subunit vaccines to assess their immunogenicity and protective efficacy (Zeng et al., 2015; Malfertheiner et al., 2018). The robust CD4^+^ T cell-mediated immunity in this model, a critical correlate of vaccine protection, has been thoroughly validated (Flach et al., 2011; Ghasemi et al., 2022; Sayi et al., 2009). For instance, studies by Xie et al. demonstrated that an oral vaccine significantly increased specific serum IgG and mucosal sIgA levels, accompanied by the secretion of cytokines such as IFN-γ (Th1), IL-4 (Th2), and IL-17 (Th17) (Pan et al., 2018). In contrast, C57BL/6 mice are more commonly used for evaluating inactivated vaccines, such as the LT R192G vaccine (Sjökvist Ottsjö et al., 2013) and whole-cell + α-GalCer vaccine (Longet et al., 2019). Research by Song et al. showed that the use of outer membrane vesicle adjuvants synergistically enhanced Th1/Th2/Th17 responses, with a particularly pronounced effect on Th2 and Th17 immunity (Song et al., 2020).

#### 2.2.2 Mongolian gerbil

The Mongolian gerbil serves as a pivotal animal model for studying Hp infection, effectively mimicking a broad spectrum of pathological changes that occur following human infection, such as chronic active gastritis, intestinal metaplasia, gastric ulcer, and gastric cancer (Sawada et al., 1999). The unique value of the Hp-infected gerbil model lies in its dynamic pathological progression: the unique value of this model is underscored by its dynamic pathological progression: the early stage of infection is marked by acute inflammation and a notable increase in apoptosis, which elucidates the initial pathogenic mechanisms of Hp (Lin et al., 2024a). Meanwhile, the precancerous stage, characterized by a high incidence of intestinal metaplasia and dysplasia around 26 weeks, provides a critical opportunity to validate oncogenic pathways, such as abnormalities in CDX1/2 (Naumann et al., 2023). Meanwhile, gerbils have also been used to develop intervention strategies, including multivalent epitope-based vaccine CFAdE, which has been shown to significantly reduce Hp colonization and alleviate gastritis by activating the sIgA mucosal barrier, IgG humoral immunity, and eliciting a mixed CD4^+^ T-cell response (Guo et al., 2017). However, the widespread application of this model is constrained by the prolonged experimental duration (12–24 months required to induce carcinogenesis), the high costs associated with feeding, and the genetic heterogeneity due to the absence of inbred lines (Zhang et al., 2016).

#### 2.2.3 Rats

In recent years, the utilization of rat models in Hp research has seen a notable increase due to their high susceptibility to this bacterium. Werawatganon et al. employed Sprague-Dawley rats that were pre-treated with streptomycin and subsequently inoculated with Hp bacterial suspensions via gavage, achieving long-term colonization of the stomach and inducing mild to moderate gastritis (Werawatganon, 2014). Concurrently, Hispid Cotton rats are capable of developing persistent colonization (lasting at least 38 weeks) following Hp infection, which induces chronic active gastritis primarily in the gastric sinus, along with pathological changes (Elseweidy et al., 2010) and immune responses analogous to those observed in human infections (Mähler et al., 2005). Furthermore, in an ischemia-reperfusion-induced gastric ulcer model, Hp infection markedly delayed ulcer healing and intensified the inflammatory response (Konturek et al., 2000).

Rat models have also been widely used in drug efficacy research. For instance, Zuojin pills improved Hp-induced gastric epithelial cell damage in an SD rat model of chronic atrophic gastritis (Wen et al., 2022). Similarly, the SD rat model of Hp-induced chronic atrophic gastritis was used to investigate the anti-inflammatory activity of jatrorrhizine and the related signaling pathways (Xie and Yao, 2025).

#### 2.2.4 Model selection strategies and research applications

In vaccine development research, initial immunogenicity evaluations often rely on mouse models due to their well-defined immune background and operational convenience (Pan et al., 2018; Longet et al., 2019; Kotloff et al., 2001). However, infection in these models typically only induces mild gastritis, failing to adequately mimic the disease progression observed in human infections. This limitation results in poor translation of preclinical data to clinical outcomes (Sutton and Boag, 2019; Sutton and Doidge, 2003). If the goal is to observe disease progression and severe pathological changes, the gerbil model is more appropriate (Naumann et al., 2023; Zhang et al., 2016). Non-human primate and beagle dog models offer higher clinical translatability due to their ability to more accurately simulate human gastric physiology and immune responses. However, their widespread application is limited by high husbandry costs, operational complexity, and poor colonization stability (Dubois et al., 1998; Rossi et al., 2004).

Mouse and rat models are commonly used to evaluate the efficacy of drugs. Currently, several synthetic antibacterial agents against Hp that have shown efficacy in mouse or rat experiments have successfully entered the clinical trial phase. For example, *in vivo* anti-infection experiments showed that rifaquizinone (TNP-2092), a multi-targeting conjugate molecule that exerts bactericidal effects by simultaneously inhibiting RNA polymerase, DNA gyrase, and topoisomerase IV, alone significantly reduced gastric Hp colonization in mice, with an effect comparable to standard triple therapy (Nazli et al., 2022). In terms of pharmacokinetics, another drug, rifasutenizol (TNP-2198), which acts through a synergistic mechanism involving the inhibition of RNA polymerase and the activation by nitroreductase to produce highly reactive species, exhibited superior pharmacokinetic characteristics and higher oral bioavailability compared to TNP-2092 (Ma et al., 2022). Furthermore, studies conducted in rat models revealed that SQ-109 maintained high concentrations in the stomach 4 h after oral administration (Makobongo et al., 2013). However, significant differences exist between these models and humans in terms of physiological structure, infection pathology, and pharmacokinetic characteristics. Therefore, extrapolating pharmacokinetic parameters and bioavailability data obtained from rodent models to humans may lead to deviations (Nazli et al., 2022).

In gastric cancer research, the Mongolian gerbil model can spontaneously induce gastric cancer, but its main drawbacks are the long experimental duration and less than 100% carcinogenesis rate (Naumann et al., 2023; Hurtado-Monzón et al., 2024; Zhang et al., 2024). Additionally, several knockout or transgenic mouse models, such as insulin-gastrin (INS-GAS) (Wang et al., 2000), interferon-gamma (IFN-γ) and tumor necrosis factor-alpha (TNF-α) knockout (Yamamoto et al., 2004), IL-10 knockout (Berg et al., 1998), Fas antigen transgenic (Cai et al., 2005), p27 deficient (Kuzushita et al., 2005), and CagA transgenic mice (Ohnishi et al., 2008) are also prone to developing gastric cancer when administered a high-salt diet or chemical carcinogens in conjunction with Hp infection (Pritchard and Przemeck, 2004). However, these models often bypass the stepwise progression typically seen in human disease.

### 2.3 Pre-treatment

Studies have demonstrated that Hp can colonize for extended periods in germ-free and immunodeficient mice, whereas it survives only briefly in normal mice. This disparity is attributed to the presence of dominant flora, such as *Lactobacillus*, in the stomachs of normal mice, which forms protective biofilms that maintain microenvironmental homeostasis and inhibit Hp colonization. In contrast, the absence of normal flora in germ-free mice and the imbalance of flora in immunodeficient mice fail to provide effective defense against Hp colonization, facilitating its establishment as the dominant flora (Karita et al., 1994, 1993). Furthermore, multiple studies have confirmed that antibiotic pre-treatment markedly enhances the susceptibility of experimental animals to Hp by disrupting the normal flora of the gastrointestinal tract (Kong et al., 2022). Ma et al. (2020) demonstrated that treating BALB/c mice with a combination of azithromycin, ampicillin, and gentamicin effectively eliminated the original gastric flora, leading to a significant increase in the success rate of Hp colonization to 100%. Similarly, Lv et al. (2014) confirmed successful colonization in antibiotic-pre-treated gerbils via positive urease and histopathology.

In addition, damage to the gastric mucosa occurs has been shown to increase the risk of Hp infection. When damage to the gastric mucosa occurs, the risk of Hp infection is heightened. Studies indicate that alcohol can impair the integrity of gastric mucosa and facilitate Hp adhesion and colonization (Vilaichone et al., 2013). Furthermore, certain studies have utilized drug pre-treatment to modulate the intragastric environment, thereby improving the colonization efficiency of Hp. Omeprazole (Zhou et al., 2009) effectively inhibits gastric acid secretion, while sodium bicarbonate (Liu et al., 2018) raises intragastric pH by neutralizing gastric acid, and indomethacin (Arunachalam et al., 2019) diminishes gastric mucosal defenses by inhibiting prostaglandin synthesis. Representative studies of the detection and quantification of Hp in Animal Models are presented in Table 1.

**Table 1:** Detection and quantification of Hp in animal models.

**Animals**	**Strain**	**Animal model establishment**	**Animal model identification**	**Main findings**	**References**
C57BL/6 mice	Hp SS1	0.1 mL of 10^8^ CFU/mL bacterial suspension, thrice at 72 h intervals, oral gavage	^13^C-UBT, IHC, PCR	After 8 weeks: PCR 100% positive/negative predictive values; ^13^C-UBT 90.3%/94.7%; IHC 95.5%/71.4% positive/negative predictive values.	Santos et al., 2011
C57BL/6 mice, Nrf2^−/−^ mice	Hp SS1	0.5 mL of 5 × 10^7^ CFU/mL bacterial suspension, thrice at 48 h intervals, oral gavage; high-salt diet (7.5% NaCl) for 2 months	IHC, Culture	Gastric pathology: Significant mucosal inflammation and atrophy; elevated TNF-α and IL-1β expression; enhanced Hp colonization under high-salt diet.	Yanaka et al., 2009
C57BL/6 mice	Hp SS1	0.1 mL of 10^8^ CFU/mL bacterial suspension, thrice at 24 h intervals, oral gavage	^13^C-UBT, H&E staining, qPCR	After 6 weeks: moderate inflammation (score 2) observed in 42.8% of mice.	Santos et al., 2015
C57BL/6 mice	Hp SS1	0.1 mL of 10^9^ CFU/mL bacterial suspension, twice daily at 1 h intervals for 2 days, oral gavage	FISH, Culture	After 2 weeks: Hp colonization: primarily within gastric mucus layer and gland openings; minimal free bacteria on mucus surface. Probe performance: high specificity and retention time.	Fontenete et al., 2016
Mongolian gerbil	Hp ATCC 43504	0.5 mL of 2 × 10^9^ CFU/mL bacterial suspension, every 48 h for 5 times, oral gavage	^13^C-UBT, H&E staining, IHC, Warthin-Starry Staining, Culture, RUT, Gastric fluid nested PCR	After 10 weeks: gastric mucosa exhibiting neutrophilic infiltration, chronic superficial gastritis, and atrophic gastritis. Nested PCR positivity: 100% (gastric juice and mucosa), 90% (duodenal contents), 10% (feces). UBT efficacy reduced in low-density infections.	Zhang et al., 2022
Mongolian gerbil	Hp 26695, Hp 279-a	0.5 mL of NaHCO_3_ solution for 30 min, followed by 0.5 mL of 6 × 10^8^ CFU/mL bacterial suspension, five times at 48-h intervals, monthly for 3 months; 0.2 mL of 60% ethanol solution thrice at 48 h intervals in the 4th month, oral gavage	serological test, H&E staining, Culture, RUT, PCR, Immunofluorescence	After 12 months: gastric tissues (44%) exhibiting lymphocyte aggregation, tissue regeneration, and superficial gastritis; elevated serum IL-8 levels; pancreas with no significant pathology but altered distribution of intercellular junctional proteins and actin.	Hurtado-Monzón et al., 2024
Mongolian gerbil	Hp ATCC 43504	0.5 mL of 2 × 10^9^ CFU/mL bacterial suspension, every 48 h for 5 times, oral gavage	H&E staining, IHC, Immunofluorescence	Initial stage (5–35 weeks): Elevated expression of inflammatory factors, mild to moderate gastritis. Later stage (40–90 weeks): decreased expression of inflammatory factors, reduced gastritis, development of Hp-induced immune tolerance.	Zhang et al., 2024
Swiss mice	Hp SS1	0.1 mL of 1.2 × 10^7^ CFU/mL bacterial suspension, orally via polyethylene catheter	H&E staining, Warthin-Starry staining, Giemsa, ELISA, Culture, RUT	After 16 weeks: mild chronic gastritis with polymorphonuclear neutrophil infiltration and submucosal lymphoid follicle formation. Hp-specific IgM and IgG antibodies were produced within 1 week; IgG and IgA antibodies were detected in serum, gastric contents, and saliva by week 16.	Ferrero et al., 1998
BALB/c mice	Hp CPY2052	Single oral gavage of 10^8^ CFU/mL bacterial suspension using stainless steel catheter	H&E staining, IHC	After 2 weeks: 50% (2/4) infection through fecal contact transmission.After 4 weeks: 70% (7/10) infection; mild to moderate gastritis in gastric mucosa of infected mice.	Yoshimatsu et al., 2000
INS-GAS mice	Hp PMSS1	2 × 10^9^ CFU/mL bacterial suspension, every 48 h for 5 times, oral gavage	H&E staining, IHC, PCR	Infected male mice displayed evident inflammatory cell infiltration and intestinal metaplasia in the gastric mucosa.	Peng et al., 2025

A search was conducted in the PubMed database using the query “(*Helicobacter pylori*[Title]) AND (mice[Title]) OR (gerbil[Title]) AND (animal model)” to retrieve literature from the past 30 years.A total of 476 relevant articles were initially screened. After excluding studies with highly repetitive modeling schemes, detection methods, and primary pathological results, a final total of 10 articles were included for this comparative analysis.

## 3 Detection and quantification of Hp in animal models

**Figure 2:**
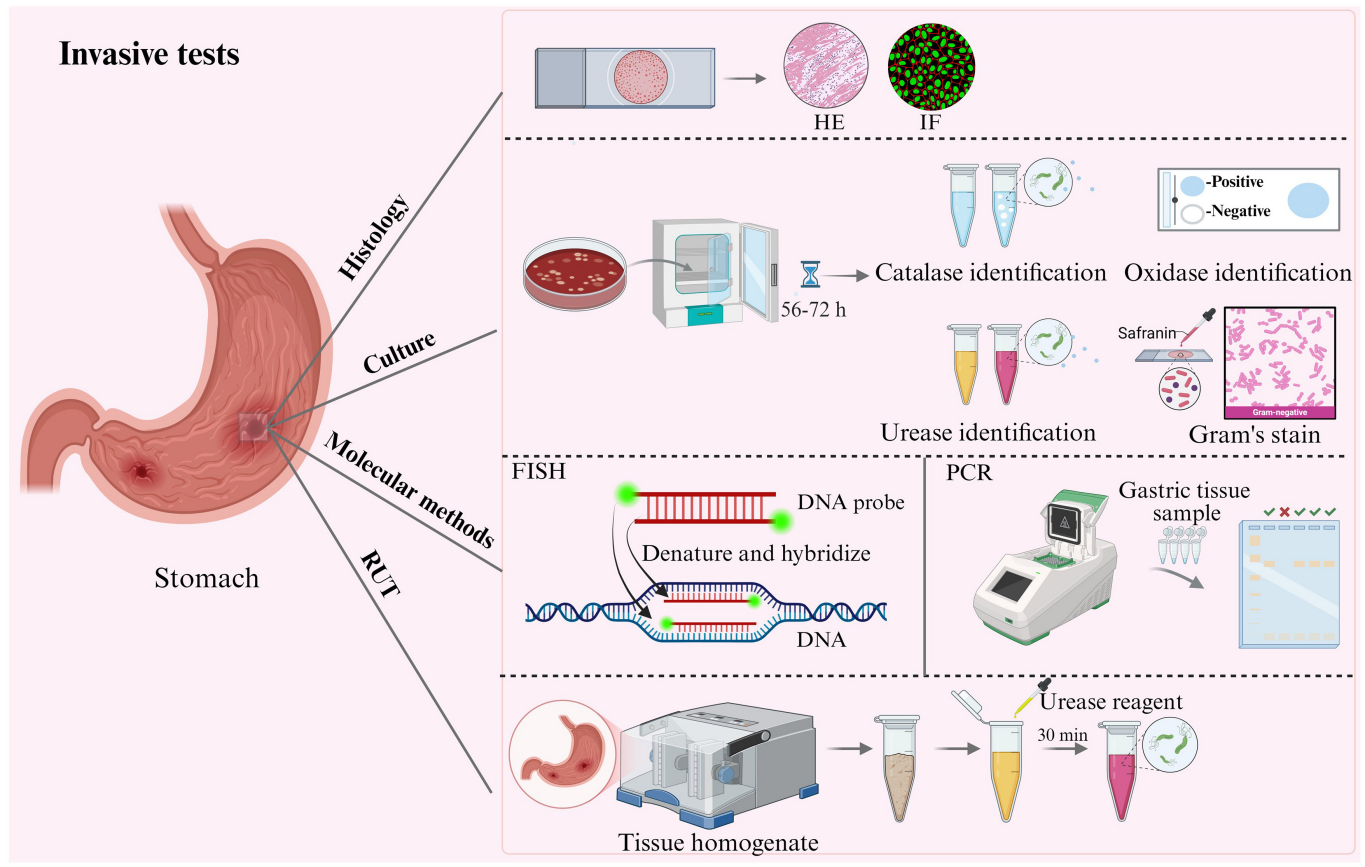
Schematic diagram of invasive tests in animal models of Hp infection. Invasive tests, including histology, culture-based methods, molecular detection and rapid urease test.

**Figure 3:**
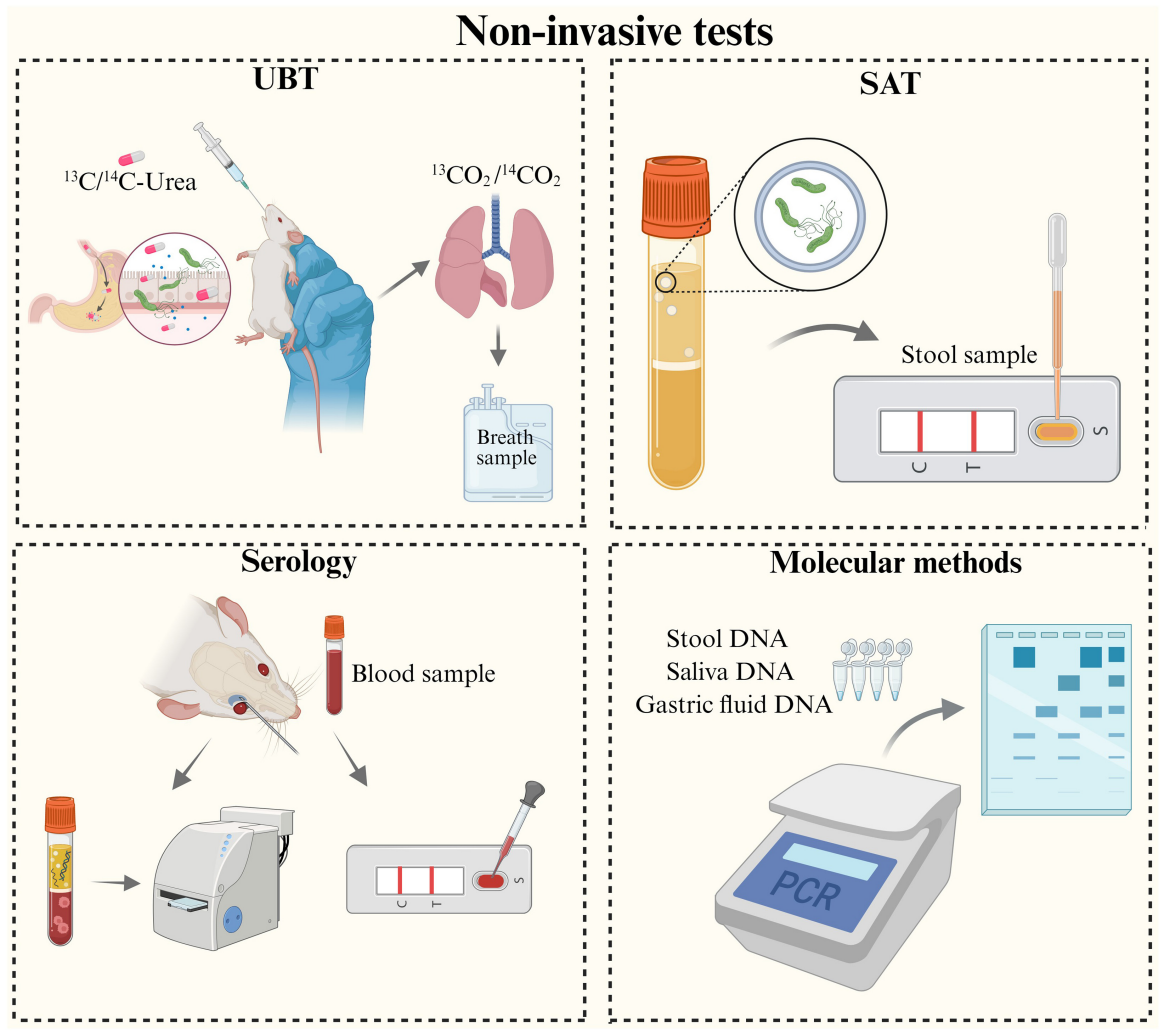
Schematic diagram of non-invasive tests for animal models of Hp infection. Non-invasive tests, including the urea breath test (UBT), stool antigen test (SAT), serologic tests, and molecular detection.

Accurate identification of Hp infection is a crucial prerequisite for studying its pathogenic mechanisms, the progression of gastric lesions, and the evaluation of vaccine efficacy. Currently, Hp detection techniques are categorized into two main types: invasive (Figure 2) and non-invasive tests (Figure 3). Invasive tests including histology, culture-based methods, rapid urease test (RUT) and molecular detection (gastric tissue). Non-invasive tests, including the urea breath test (UBT), stool antigen test (SAT), serologic tests, and molecular diagnostics (DNA from stool, saliva, gastric fluid).

### 3.1 Invasive tests

#### 3.1.1 Histology

Histological examination of gastric mucosal biopsies is considered the gold standard for characterizing Hp-induced pathology. Its diagnostic accuracy depends critically on biopsy site selection and staining protocol (Braden, 2012). Multi-site biopsies (antrum, body, greater curvature) significantly improve Hp detection sensitivity. Notably, biopsies from the gastric body's greater curvature demonstrate superior sensitivity for specific conditions like bleeding peptic ulcers, atrophic gastritis, intestinal metaplasia, and gastric cancer (Lee and Kim, 2015).

Currently, the commonly used staining methods for tissue sections include routine hematoxylin-eosin (H&E) staining, Giemsa staining, Warthin-Starry silver staining, and immunohistochemical (IHC) staining. In histological evaluation, these techniques differ significantly in principle and application: H&E staining is a routine and fundamental method that provides a comprehensive overview of tissue architecture, inflammatory cell infiltration, and host pathological changes (Patel et al., 2014). Both Giemsa staining and Warthin-Starry silver staining enhance the contrast between Hp microorganisms and the background. Giemsa staining characteristically colors Hp a deep bluish-purple and is relatively low-cost (Rotimi et al., 2000; Laine et al., 1997), whereas Warthin-Starry silver staining imparts a brownish-black color to the bacteria against a yellow background, creating a strong visual contrast (Doglioni et al., 1997). IHC employs specific antibodies targeted against Hp antigens, offering the highest specificity and sensitivity. It enables accurate detection even in cases of low bacterial density, poor staining quality, or when Hp assumes coccoid forms (Hartman and Owens, 2012).

Animal studies utilizing staining methods have elucidated the pathological mechanisms associated with Hp. Fu et al. (2024) and Jiang et al. (2021) employed H&E and IHC staining in Hp SS1-infected mice, revealing a progression from acute to chronic inflammation, mucosal atrophy, and elevated expression of proliferation markers such as proliferating cell nuclear antigen and Ki-67. Furthermore, Zhang et al. (2016) combined H&E, Warthin-Starry staining, and urease testing in a Mongolian gerbil model, demonstrating that 71.4% of gerbils in the Trx1 overexpression group had developed well-differentiated tubular adenocarcinoma by the 90th week post-infection.

Recently, researchers have continued to explore novel histological methods and improve staining techniques. Fan et al. (2020) developed a modified Giemsa staining method that achieves comparable accuracy to the traditional method while being less time-consuming and more cost-effective by altering the staining solution and procedures. Additionally, Akashi et al. (2019) proposed a new approach using gGlu-HMRG, a γ-glutamyltranspeptidase-activatable fluorescent probe. Rapidly activated by Hp-associated γ-glutamyl transpeptidase, it turns from a non-fluorescent precursor to a strong green fluorescent signal in 5–15 min. The probe was applied for *ex vivo* detection of clinical samples, tested on gastric antrum and body biopsies from 46 patients. The sensitivity and specificity of this method were 75.0% and 83.3% in the gastric antrum, and 82.6% and 89.5% in the gastric body, respectively, with a detection time of only 15 min, enabling rapid differentiation of Hp-positive, negative, and post-eradication specimens. Its good performance in clinical samples supports its potential for use in Hp-infected animal models. For example, it could dynamically track Hp colonization, diffusion and quantity in animal gastric mucosa via real-time fluorescence imaging.

#### 3.1.2 Culture-based methods

The primary application of culture-based methods in animal models is to quantify the bacterial load and to isolate strains for downstream analysis. Although culture demonstrates 85–95% sensitivity, a value lower than that of histological examination or rapid urease tests, it achieves 100% specificity and enables downstream applications such as antimicrobial resistance profiling for fluoroquinolones and clarithromycin, validation in animal models, and molecular analyses (Pohl et al., 2019; Guevara and Cogdill, 2020; Graham and Miftahussurur, 2018).

As a fastidious microaerophile, Hp requires blood or serum-supplemented enrichment media such as Columbia agar, brain heart infusion agar, or Brucella agar for reliable isolation from complex samples like gastric tissue (Hazell et al., 1989). In animal studies, accurate quantification of gastric colonization levels is crucial. Liu et al. tested Hp SS1, revealing that a colony count of 2.0 × 10^1^ CFU/mL was sufficient to culture positive clone (Liu et al., 2022). However, the sensitivity of the culture decreased when the same concentration of Hp bacterial solution was added to gastric tissues compared to the Hp bacterial solution alone. This reduction in sensitivity may be attributed to contamination from normal flora present in gastric tissues, which inhibits the growth of Hp (Peretz et al., 2015; Miendje Deyi et al., 2010).

To improve the recovery and quantification of Hp from animal gastric tissues, it is highly recommended to dilute the gastric homogenate before plating and to use selective antibiotic supplements (e.g., vancomycin, amphotericin B, cefsulodin, trimethoprim) in the culture media (Patel et al., 2014; Hazell et al., 1989). Additionally, sampling high bacterial density areas such as the gastric antrum and fundic mucosa can further enhance the accuracy of colonization assessment (Langner et al., 2009).

#### 3.1.3 Rapid urease test

The RUT is a standard method for detecting active Hp infection, exploiting the organism's potent urease activity. Hydrolysis of urea produces ammonia, inducing a pH shift detected visually by color change in indicators like phenol red (Uotani and Graham, 2015). In animal models, RUT is typically applied directly to fresh gastric mucosal tissues, including samples from the gastric antrum or gastric body of mice and Mongolian gerbils. It has become a preferred tool for primary screening of infections due to its ease of use, yielding results within 30 min to 24 h, and its cost-effectiveness (Lee et al., 1997). However, RUT sensitivity is constrained by a bacterial load threshold; false negatives can occur during early infection or post-treatment when bacterial loads are low. False positives may arise from urease-producing commensal gastric *Helicobacter* species (e.g., *H. hepaticus*). Furthermore, Hp infections in treated animals often exhibit focal distribution at low bacterial loads. Restricting biopsy sampling to a single site risks misdiagnosis due to missing lesions or interference by commensal bacteria (Malfertheiner et al., 2017).

To enhance the reliability of the RUT in animal models, several optimization strategies have been proposed by researchers. Celik et al. (2021) developed a natural pH indicator based on red cabbage anthocyanins (RCE@test), which can detect Hp at concentrations as low as 10 CFU/mL and 1 CFU/mL within 15 min and 3 h, respectively. This method demonstrates superior sensitivity compared to the conventional phenol red method. Furthermore, combining multiple assays, such as histopathology (e.g., Warthin-Starry staining) or PCR techniques, can significantly enhance diagnostic accuracy. Although the RUT does not facilitate strain typing or quantitative analysis, its rapidity and low cost render it indispensable for large-scale infection screening in animal models.

### 3.2 Non-invasive tests

#### 3.2.1 Urea breath test

The UBT is the gold standard for non-invasive Hp detection in humans and post-treatment monitoring, measuring exhaled labeled CO_2_ produced by bacterial urease hydrolysis of ingested ^13^C or ^14^C urea. A study conducted by Gisbert and Pajares (2004) demonstrated that the sensitivity and specificity of UBT surpassed 90% in human subjects. However, extending UBT applications to animal models necessitates species-specific adaptations due to physiological differences. This enables dynamic monitoring of Hp infection without the need for euthanizing the animals. The ^13^C-UBT method developed by Santos et al. (2011) successfully enabled real-time monitoring of the infection process in a 2-month mouse model. Using PCR as the reference standard, this method achieved a sensitivity of 96.6% and a specificity of 85.7%. Furthermore, Zhang et al. (2022) designed a ^13^C-UBT device tailored for gerbils, establishing the detection method and threshold for this species. Their findings revealed that 50% of the gerbils were infected with Hp within 85 weeks post-infection.

Although the ^13^C-UBT has been successfully adapted for real-time monitoring of Hp infection in animal models such as mice and gerbils (Santos et al., 2011; Zhang et al., 2022), it is important to note that this method is not yet established as a standard practice in most research settings involving Hp animal models. Unlike humans, experimental animals have distinct physiology and environments, potentially affecting test accuracy. Key factors affecting the accuracy include animal fasting duration and potential exposure to feces or residual feed (Santos et al., 2011; Zhang et al., 2022). Animal bulimia may cause false positives in ^13^C-UBT via fecal urease-positive bacteria; fasting exposure to food residue or fur can also do so due to dietary urease or fecal contamination (Hammond et al., 1999). Santos et al. (2011) determined that a fasting duration of 14 h is optimal, noting that false-positive results occurred when the fasting period was either shortened or extended, influenced by diet, feces, or rat pelts.

#### 3.2.2 Stool antigen test

As a non-invasive approach, the stool antigen test detects Hp-specific antigens like urease and CagA using monoclonal antibodies, enabling dynamic infection monitoring and treatment evaluation in animal models. Moon et al. (2013) used an HpSA assay (SD Bioline kit) in mice model, showing 83.33% sensitivity for live-animal monitoring thereby confirming its utility in monitoring the infection status of living animals. Similarly, Lee et al. (2023) validated Hp eradication in a C57BL/6 mouse model of infection using the Asan Easy Test^®^ kit. Their findings, combined with histological analysis, revealed that clover leaf extract significantly attenuated the chronic inflammatory response induced by Hp colonization of gastric epithelial cells through the inhibition of pro-inflammatory cytokines (e.g., IL-6, TNF-α).

Despite the operational convenience of conventional enzyme-linked immunosorbent assays (ELISA) and immunochromatographic techniques, their sensitivity and specificity are often compromised by the cross-reactivity of animal intestinal commensals, low bacterial loads, and physiological differences in the gastrointestinal tract (Stark et al., 2024). To address these limitations, technological innovations have become a focal point of research in recent years. Kubo et al. (2024) developed a new bioluminescent enzyme immunoassay (BLEIA). This method combines a highly efficient luciferase system with catalase-targeting monoclonal antibodies and incorporates an optimized low-background interference design. Although BLEIA uses a Cut-off Index for result interpretation and is classified as semi-quantitative, its performance is significantly superior to that of conventional techniques. Thanks to these innovative design features, BLEIA effectively overcomes critical limitations of traditional semi-quantitative methods, such as cross-reactivity and interference from low bacterial loads, thereby achieving improved sensitivity and specificity.

#### 3.2.3 Serologic tests

Hp expresses key virulence factors including CagA, VacA, and urease subunits UreA/UreB. These antigens stimulate host antibody production, forming the basis for serological detection methods that primarily target IgG antibodies (Costa et al., 2024). In a study utilizing a guinea pig model, Gonciarz et al. (2020) demonstrated that serum levels of anti-Hp IgM and IgG antibodies were significantly elevated in Hp-infected guinea pigs, as indicated by serologic tests. This was accompanied by increased levels of C-reactive protein and decreased levels of tumor necrosis factor (TNF), suggesting a robust immune response and inflammatory reaction triggered by Hp infection. Kuo et al. (2007) used STAT-PAK to detect Hp infection in Mongolian gerbils, achieving a success rate of 88% in 50 inoculated subjects, with a sensitivity of 90.9% and specificity of 100%. However, the negative predictive value was only 60%. This indicates that in the early stages of infection, the antibody levels produced in the animal's body may be insufficient for detection, leading to false negative results (Butt et al., 2023).

Despite this limitation in early stages of infection, serological detection holds significant value in vaccine research, as it facilitates the evaluation of vaccine immunogenicity and protective efficacy in animal models. In studies on Hp-targeted vaccines (Pan et al., 2018; Yu et al., 2010; Ansari et al., 2021; Liu et al., 2019), the levels of vaccine-induced immune responses and their associated protective potential were assessed by measuring the titers of specific antibodies (e.g., IgG, IgM) in serum. The significant rise in antibody titers following immunization serves as crucial evidence for successful vaccine-induced humoral immune responses.

### 3.3 Molecular detection

#### 3.3.1 PCR

PCR technology has been extensively employed for the identification of pathogens in Hp infection models due to its high sensitivity and versatility. While traditional gastric mucosal tissues obtained through dissection remain the gold standard for direct assessment of gastric colonization, recent research has increasingly favored non-invasive or semi-invasive sampling strategies. For example, fecal samples allow for non-invasive monitoring of infection dynamics and therapeutic efficacy by detecting shed bacterial components or DNA fragments (Zhang et al., 2022; Moon et al., 2013).

Despite their advantages, fecal samples remain underutilized in animal model studies due to various technical limitations. Issues such as bacterial degradation, host DNA interference, and low microbial biomass in feces often compromise detection sensitivity (Sun et al., 2022; Yan et al., 2019). Yoshimatsu et al. (2000) investigated fecal-oral transmission in nude mice through PCR analysis of saliva and fecal samples. Their study revealed a 70% transmission rate following 4 weeks of fecal exposure, but also exposed the inherent limitation of conventional PCR in achieving consistent sensitivity.

Recent technological innovations have begun to address these limitations. Talarico et al. (2016) developed a fecal-based microdroplet digital PCR (ddPCR) assay targeting the 16S rRNA and CagA virulence genes of Hp. Using microdroplet partitioning, this method achieved 10 copies/μL sensitivity in low-biomass samples by reducing background interference. Unlike quantitative PCR (qPCR), ddPCR offers superior inhibitor tolerance and absolute quantification of infection severity, establishing a standardized framework for fecal analysis in animal models. Further applications of PCR in characterizing Hp infection models are summarized in Table 2.

**Table 2:** PCR detection of Hp infection in animal models.

**Animals**	**Srains**	**Samples**	**PCR/primers**	**Timing of colonization and positivity rate**	**References**
Mongolian gerbil	Hp ATCC 43504	Gastric fluid, gastric tissue, duodenal content, colonic stool	Nested PCR; Ure A	10 weeks; positivity rate: 100% for gastric tissue, 90% for duodenal contents, 10% for colonic feces	Zhang et al., 2022
C57BL/6 mice	Hp SS1	Gastric mucosa	Nested PCR; 16s, 26-kDa, Vac A, Cag A	2 and 8 months; positive rate: 93.3%	Huang et al., 2009
BALB/c mice	Hp SS1	Gastric mucosa	RT-PCR; TLR4, Foxp3	12 weeks; Hp eradication rate: 58.33%	Gong et al., 2015
C57BL/6 (p27^−/−^) mice	Hp SS1	Gastric tissue	RT-PCR; SSA	60 weeks; 83.3% presented with significant heterogeneous gastric hyperplasia or cancer	Kuzushita et al., 2005
Mongolian gerbil	Hp 26695	Gastric tissue, pancreas	PCR; glmM, Cag A	12 months; positivity rate: 33%	Hurtado-Monzón et al., 2024
C57BL/6J, C57BL/10J, BALB/c, CB6F1 mice	Hp SPM326, Hp M1.16	Gastric tissue	RT-PCR; 16S, cag A	2–10 weeks; positivity rate: SPM326 (76%); M1.16 (89%)	Smythies et al., 2000
BALB/c mice	Hp CPY2052	Gastric tissue, stool	PCR; Ure B, Ure A	2–4 weeks; positivity rate: 100%	Yoshimatsu et al., 2000
Swiss mice	Hp SS1	Gastric tissue	PCR; 16S	2–4 weeks; positivity rate: 100%	Suganya et al., 2017
C57BL/6 mice	Hp SS1	Gastric tissue	RT-PCR; 23S	18 weeks; positivity rate: 100%	Santos et al., 2015

Using the PubMed database, we conducted a search with the query “(Helicobacter pylori) AND (mice) AND (PCR)”, resulting in a preliminary selection of 284 articles. After excluding studies with duplicate model establishment methods and repetitive PCR primers, a total of nine articles were included for this comparative analysis.

#### 3.3.2 Fluorescence in situ hybridization

Fluorescence *in situ* hybridization (FISH) is a molecular diagnostic technique that employs fluorescent probes for the specific detection of Hp, typically necessitating gastric biopsy samples for fluorescent labeling and analysis (Azevedo et al., 2019; Amann and Fuchs, 2008). Fontenete et al. (2015) developed a Cy3-labeled LNA2′OMe probe (HP_LNA/2OMe_PS) targeting the Hp 16S rRNA gene, optimizing hybridization time and pH conditions to complete FISH within 30 min while preserving high sensitivity and specificity across a pH range of 2 to 7. Subsequent research by the same group (Fontenete et al., 2016) utilized FISH in a C57BL/6 mouse model, successfully identifying mucus-adherent Hp SS1 strains colonizing the gastric epithelium. The LNA probe exhibited exceptional cytocompatibility and stability, facilitating specific Hp detection in extremely acidic environments in murine gastric mucosa with minimal background noise. Kao et al. (2022) developed a microfluidic digital droplet FISH platform utilizing locked nucleic acid LNA/DNA molecular beacons. This innovation facilitates single-cell-level quantification within 1.5 h, providing a rapid and robust solution for bacterial analysis in heterogeneous samples.

Further studies have elucidated the complex colonization behavior of Hp within gastric niches. Sigal et al. (2015) employed FISH to reveal that Hp not only colonizes the superficial mucus layer but penetrates deep into gastric glands, forming microcolonies concentrated in the mid and basal regions. These microcolonies directly interact with proliferating progenitor and stem cells. Additionally, FISH-based analyses in murine models demonstrated that chronic Hp-induced inflammation promotes the migration of bone marrow-derived cells to the gastric mucosa, where they differentiate into epithelial cells. This process is linked to adenopathic progression, including mucoepidermoid metaplasia, pseudointestinal metaplasia, and dysplasia (Varon et al., 2012).

### 3.4 Single-cell RNA sequencing and spatial transcriptomics

Single-cell RNA sequencing (scRNA-seq) is a powerful tool for resolving gene expression at the individual cell level, significantly advancing our understanding of cellular heterogeneity and dynamic changes in the microenvironment. For instance, Hu et al. (2025) utilized this technology to reveal that Hp remodels the gastric immune microenvironment through multiple mechanisms, including suppressing antigen presentation, driving macrophage dysfunction, limiting T-cell clonal expansion, and activating immune checkpoints. Meanwhile, Li et al. (2025) constructed a single-cell atlas of the progression from Hp-associated gastritis to gastric cancer, uncovering cellular heterogeneity and microenvironmental evolution throughout this process. They particularly observed abnormal enrichment of enterocyte cells from precancerous lesion gastrointestinal metaplasia. Additionally, scRNA-seq analysis revealed significant upregulation of DUOX2 and DUOXA2 gene expression in gastric crypt cells following Hp infection (Hofer et al., 2025).

However, a inherent limitation of scRNA-seq is that it requires tissue dissociation into single cells suspensions, resulting in the loss of spatial context. Spatial transcriptomics overcomes this by capturing gene expression information from specific locations while preserving tissue architecture (Molla Desta and Birhanu, 2025; Crosetto et al., 2015). Using this technology, Lin et al. (2024b) observed significantly higher ONECUT2 expression in Hp-infected tissues compared to uninfected controls, with spatial colocalization between ONECUT2 and RELA, a key NF-κB pathway gene. Moreover, regions expressing high levels of stem cell markers (e.g., CD44 and SOX9), the proliferation marker Mki67, and the Wnt/β-catenin target gene CCND1 largely overlapped with the ONECUT2 high-expression areas. It is precisely due to this core advantage of preserving spatial information that spatial transcriptomics is believed to have great potential in studying Hp infection animal models. It could not only pinpoint infection-enriched regions and specific cell types but also visualize the infiltration sites of immune cells, thereby potentially providing crucial evidence for more accurately assessing disease progression and predicting prognosis.

## 4 Conclusion and prospect

Animal models of Hp infection are fundamental for elucidating pathogenesis, evaluating vaccines, and developing new therapies. Since the initial isolation of Hp from human gastric mucosal tissues, Various species have been utilized, contributing significantly to research. Both strain selection and infection protocols are also continuously being optimized to improve infection rates and stability. Optimization strategies include using clinical isolates with complete pathogenicity islands, serial *in vivo* passage for host adaptation, matching animal species to strain characteristics, and refining infection parameters to enhance the sensitivity of animal model to Hp infection and better replicate human infection dynamics. Detection methods in models differ partially from clinical practice; traditional techniques remain prevalent, while emerging molecular techniques and spatial transcriptomics are gaining increasing attention. These advances in detection methods lay a robust foundation for in-depth studies on the epidemiology of Hp and the effective prevention and eradication of this pathogen.

However, the establishment of animal infection models primarily faces the challenge of low efficiency in translating preclinical data into clinical outcomes. One major factor is the complex immune evasion mechanisms formed over millennia of co-evolution between the pathogen and its human host. On the other hand, there is the poor stability of infection animal models, largely due to interspecies differences in gastric microenvironments and immune responses, which often leads to diminished pathogenicity of human-derived Hp strains in animal hosts. For example, mouse models replicate acute inflammation but rarely progress to gastric cancer, while gerbils can develop adenocarcinoma, though with long latency and high variability. Monkeys and cats closely mimic human pathology but are naturally Hp-infected, which may compromise experimental reliability. Moreover, no unified detection “gold standard” exists for Hp detection in animal models. Non-invasive methods such as the UBT are limited in rodents due to rapid metabolism and variable carbon dioxide levels, while SAT are hindered by antigen degradation and matrix complexity. Invasive techniques like histology and culture offer high specificity but require terminal sampling and are prone to false negatives with low bacterial loads. Molecular tools provide sensitive detection and resistance profiling, yet cannot distinguish live from dead bacteria and remain technically demanding and costly. These advances lay a foundation for epidemiological studies and the effective prevention and eradication of this pathogen.

Therefore, we still face the dual challenges of establishing Hp infection animal models and subsequently detecting and quantifying Hp in these models. The limitations of traditional models drive exploration of new paradigms: Gastric organoids provide humanized 3D platforms simulating gastric mucosa, enabling study of Hp colonization, virulence mechanisms, and immune cell co-culture-induced inflammation, and successfully replicating chemotactic migration and CagA-mediated epithelial damage (Kim et al., 2022; Krishnamurthy et al., 2022) Humanized immune system mouse models integrate human immune responses (Chen et al., 2021); CRISPR gene editing enables mechanistic studies through targeted modification of host and pathogen genes, exemplified by Mongolian gerbil Cystatin C and Apolipoprotein A-II knockout models for Hp infection (Almeida et al., 2022; Wang et al., 2020). However, the development of stable animal models that mimic human infection and disease progression, along with the establishment of accurate and sensitive standardized detection systems, requires further research. This review provides a comprehensive overview of the key elements involved in establishing Hp infection animal models and summarizes recent advances in model detection techniques. The aim is to provide a practical reference for establishing stable, reproducible, and highly human-relevant animal models that support the preclinical evaluation of novel therapeutic agents and candidate vaccines.
